# Patient-Centered Podcasts: An Educational Innovation to Improve Attitudes Toward Patients with Opioid Use Disorder Among Internal Medicine Practitioners

**DOI:** 10.1007/s11606-026-10222-y

**Published:** 2026-01-29

**Authors:** Joshua Onyango, Chase Webber, Mario Davidson, Helen Cai, Katherine A. Gielissen, Charlene M. Dewey

**Affiliations:** 1 Department of General Internal Medicine and Public Health, Vanderbilt University Medical Center (VUMC), Nashville, TN USA; 2 Department of Biostatistics, VUMC, Nashville, TN USA; 3 Yale University School of Medicine, New Haven, CT USA; 4 Department of Medicine and Pediatrics, Emory University School of Medicine, Atlanta, USA

**Keywords:** patient-voice, stigma, attitude, empathy, podcasts, opioid use disorder, instructional design

## Abstract

**Background:**

Despite the high prevalence of substance use disorder (SUD) in primary care and hospital settings, few easily deployable interventions exist to address stigma and empathy decline among general internists.

**Aim:**

To develop and pilot the first patient-centered podcast to improve attitudes toward opioid use disorder (OUD) patients among internal medicine residents and faculty.

**Setting:**

Academic Medical Center General Internal Medicine department.

**Participants:**

Sixty participants in needs assessment; 15 participants enrolled in a non-controlled pre-post intervention study.

**Program Description:**

We developed a novel three-episode podcast series incorporating authentic lived experience with OUD and expert commentary using a collaborative co-creation methodology. Our systematic needs assessment informed deployment of this educational innovation.

**Program Evaluation:**

Pre-post measures included attitudes (Medical Condition Regard Scale), confidence in OUD competencies, and participant satisfaction. Statistical analysis used Wilcoxon signed-rank and McNemar tests.

**Results:**

Participants demonstrated statistically significant improvement in attitude (*p* = 0.015), confidence with motivational interviewing and offering resources (*p* = 0.04).

**Discussion:**

This innovation suggests podcasts using patient voices can potentially provide attainable and scalable means to improve attitudes while addressing SUD education gaps. Larger studies are needed.

**Supplementary Information:**

The online version contains supplementary material available at 10.1007/s11606-026-10222-y.

## INTRODUCTION

In 2023 alone, approximately 105,000 peoplein the USA died of drug overdose — 76% of them involved opioids^1,2^. Physicians often stereotype people living with substance use disorder (SUD) as violent, manipulative, and with poor motivation, which leads to less engagement and diminished patient empowerment^3^. Practitioners in internal medicine are on the front line of this epidemic and need to be prepared to provide competent and non-stigmatizing care to this population.

Podcasts are a preferred learning method by learners and promote adult learning theory^4,5^. While previously existing educational podcasts have their strengths, they tend to be a repackaging of lecture-based teaching and come with many of the same shortcomings^6^. The addition of case-based learning to podcasts has the potential to optimize learning gains^7^. Directly involving patients in the learning process through storytelling, shadowing, or recorded videos has demonstrated increases in measures of empathy among medical students^8,9^.

To our knowledge, a podcast that provides case-based learning with authentic patient involvement does not exist. To address this gap, we previously developed a patient-centered podcast covering various primary care topics at Yale School of Medicine (YSM), including an opioid use disorder (OUD) series. To determine education needs at Vanderbilt University Medical Center (VUMC), we conducted a needs assessment which confirmed significant attitude and confidence gaps in OUD management.

Through this proof-of-concept pilot study, we aimed to evaluate the impact of patient-centered podcasts on internal medicine provider attitudes and confidence regarding OUD management. We sought to answer three research questions: (1) Does a patient-centered podcast improve attitudes toward patients with OUD? (2) Does the intervention increase confidence in OUD management competencies? (3) Do participants find this innovation acceptable and feasible?

## PROGRAM DESCRIPTION

**Figure 1:**
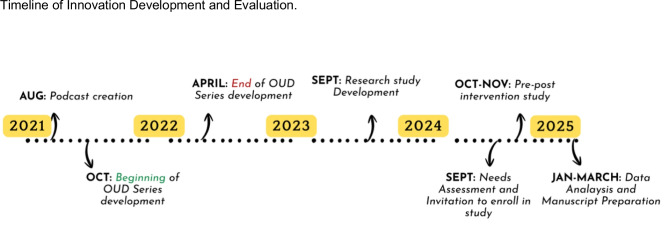
This figure illustrates the sequential development and evaluation of the patient-centered podcast innovation. Podcast creation began in August 2021 at Yale School of Medicine, with OUD-specific content development starting in October 2022 and concluding in April 2023. The evaluation study at Vanderbilt University Medical Center began with a needs assessment in September 2024 to identify educational gaps, followed by recruitment and the pre-post intervention study from October to November 2024. Data analysis and manuscript preparation were conducted from January to March 2025.

Based on gaps in education content that include patient voice, we created the first of its kind educational podcast series centering authentic lived experience for primary care education, including SUD. The development of these podcasts started in August 2021, with the creation of the OUD series specifically occurring between Oct 2021 and April 2022. To create these patient-centered podcasts, we involved Yale medical students, residents, and faculty in a collaborative co-creation process involving mentored curriculum planning, student-led patient interviews, and peer review from experts and patients. The primary challenges faced during implementation were time constraints for residents and difficulty in establishing reliable patient recruitment pipelines.The intervention in our pilot study was a three-episode OUD podcast series, averaging about 30 min each. To determine the relevance of this innovation for this population, we conducted a needs assessment among 60 internal medicine residents and faculty at VUMC in September 2024. Results confirmed significant confidence gaps in SUD management: while most felt confident with tobacco use disorder (71%), confidence dropped dramatically for alcohol (38%), opioid (35%), and stimulant use disorders (18%). One author (JO) reviewed all open-ended survey responses and identified recurring patterns through open coding. Preliminary themes were reviewed and refined through discussion with a second and third author (CD, CW) to ensure accurate representation of participant perspectives. This analysis revealed four key challenges: motivational interviewing skills, lack of desire to invest energy in SUD patients, knowledge of treatment options, and care coordination difficulties. Given the podcasts had already been developed, these findings informed our implementation, specifically targeting attitude challenges through patient integration while addressing knowledge gaps in management and resources through expert commentary (Fig. 1).

## SETTING AND PARTICIPANTS

The study participants included internal medicine practitioners at VUMC, an academic medical center in Nashville, TN, who were invited to participate in a needs assessment as described above. All the inpatient and outpatient general internal medicine faculty (*n* = 400) and categorical internal medicine residents (*n* = 150) were invited via email (see Appendix 1), and a total of 60 (around 65% female 35% male) responded, yielding a 10.9% response rate. Due to the exploratory nature of this study, we recruited a convenience sample of 15 individuals who had participated in the needs assessment. They consented and enrolled in our non-controlled pre-post pilot study through a separate REDCap link (secure online survey platform) at the end of the needs assessment survey. The study took place between October and November 2024. To incentivize participation in the pilot study, participants were entered to win one of five $50 Amazon gift cards through a raffle process. The sample consisted of 9 women (60%) and 6 men (40%). Participants represented different levels of experience: 5 residents (33.3%), 7 junior faculty (46.7%), and 3 experienced faculty (20.0%). Participants completed the intervention asynchronously and independently, accessing three podcast episodes at their convenience over 1 month via podcast streaming platforms on computers or mobile devices.

## PROGRAM EVALUATION

The needs assessment, knowledge, and confidence surveys were developed by the research team based on ACGME competencies for internal medicine. Survey items were designed to assess the six core domains of SUD care (see Appendix 1). To establish content validity, all instruments were reviewed by two faculty members with expertise in addiction medicine (CD, JO) for alignment and relevance to internal medicine practice.

We explored the preliminary impact of this educational innovation by focusing on measures of empathy, attitude, knowledge, confidence in OUD competencies, and satisfaction with listening experience. To evaluate baseline empathy among participants, we used the validated Toronto Empathy Questionnaire (TEQ), a self-reported measure for empathy with scores ranging from 0 to 64 (higher numbers symbolize higher levels of innate general empathy)^10^. This scale was only used in the pre-survey without a post-survey equivalent to capture baseline empathy and detect selection bias. No participants were excluded based on TEQ scores. The Medical Condition Regard Scale (MCRS) served as our primary pre-post outcome measure, as it is validated to assess attitudes toward patients with particular medical conditions with scores ranging from 11 to 66 (higher numbers symbolizing positive attitudes). We assessed knowledge acquisition using three multiple-choice questions (MCQ); self-reported confidence in various OUD competencies through a Likert scale; and evaluation of podcast satisfaction in the post survey.

To assess for changes in MCRS scores, knowledge, and confidence in OUD competencies, we conducted statistical analyses using R version 4.3.2. We used pairwise deletion to handle missing data, retaining all available matched pairs for each analysis. This approach allowed us to analyze changes in continuous variables (MCRS scores) using Wilcoxon signed-rank tests. We considered a *p*-value less than 0.05 statistically significant for all analyses.

For categorical variables (confidence levels), we dichotomized responses into “Lack Confidence” and “Confident” and evaluated them using McNemar’s test.

## HUMAN ETHICS AND CONSENT TO PARTICIPATE DECLARATIONS

This study was approved by the Vanderbilt University Medical Center Institutional Review Board (IRB; ID 240972). All participants provided informed consent prior to participation in both the needs assessment and the pre-post intervention study. Patient participants in the podcast creation provided informed consent for their interviews to be recorded, edited, and published.

## RESULTS

We enrolled a total of 15 participants in the study. We collected data via REDCap surveys and conducted statistical analysis using R. After applying pairwise deletion for missing data, we had a sample size of *N* = 12 (faculty = 8, residents = 4; men = 5, women = 7). The mean baseline TEQ score was 49/64 (SD = 8.8), indicating average baseline empathy among enrollees. We applied the Kirkpatrick model of program evaluation as follows while referencing our research questions (RQ).

### Kirkpatrick Level 1

#### RQ3: Do Participants Find This Innovation Acceptable and Feasible?

Participants rated the podcast highly across all evaluation dimensions. The podcast received particularly positive ratings for ease of access (75% “Strongly Agree”) and inclusion of patient voice (68.8% “Strongly Agree”). Overall satisfaction was high, with most participants agreeing or strongly agreeing with positive statements about the podcast’s listening experience (81.2%), clarity (100%), relevance (87.5%), credibility (100%), and application to practice (87.4%).

### Kirkpatrick Level 2

#### RQ1: Does a Patient-Centered Podcast Improve Attitudes Toward Patients with OUD?

**Figure 2:**
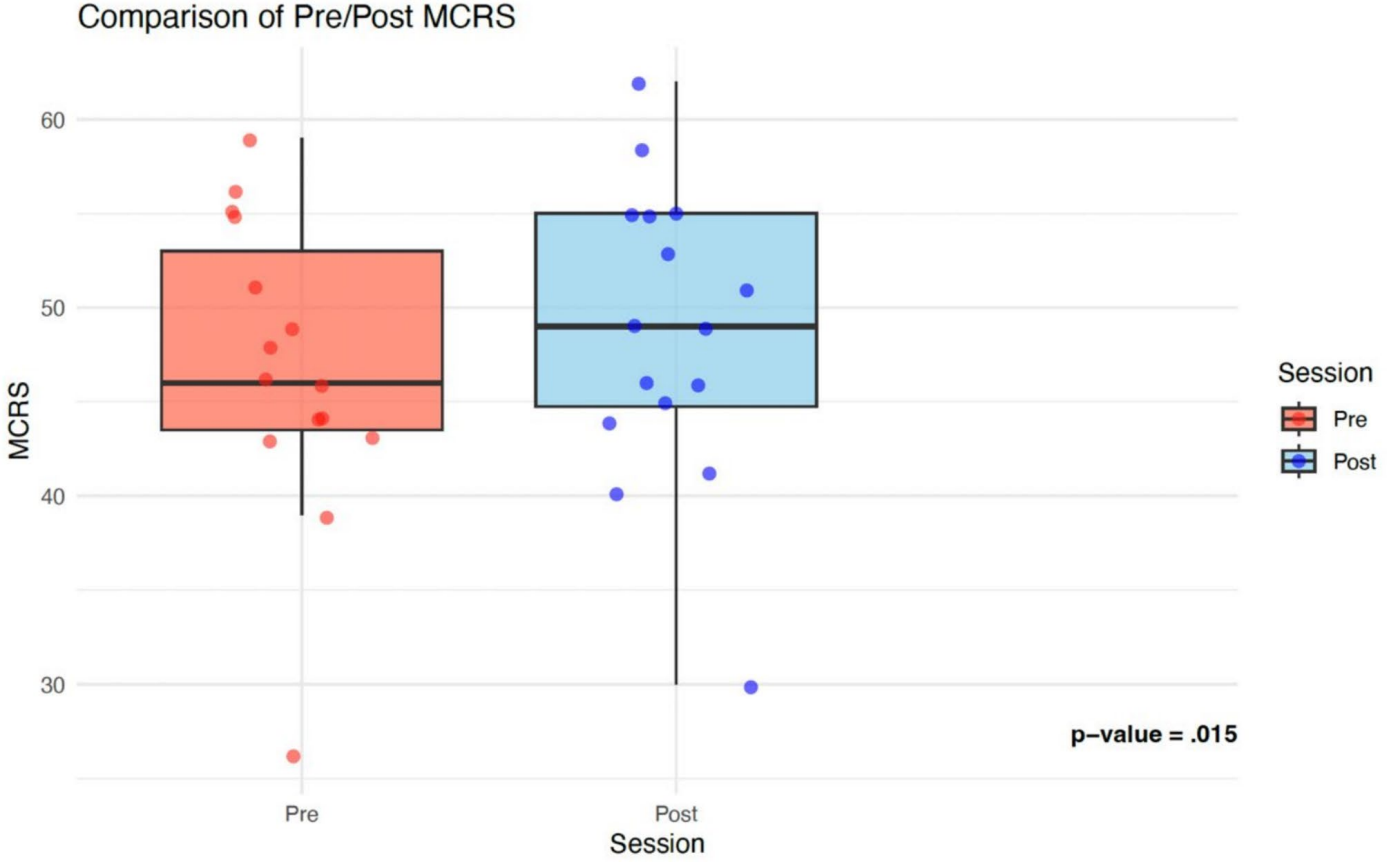
Boxplot comparison of Pre/Post Medical Condition Regard Scale (MCRS) scores. The red (Pre) and blue (Post) boxplots show individual participant responses, with median values indicated by the horizontal center line. Post-intervention scores demonstrated a statistically significant improvement compared to pre-intervention scores (*p* = 0.015).

As a result of our intervention, participants experienced a statistically significant improvement in MCRS scores from pre-intervention (mean = 46.9, SD = 9.0) to post-intervention (mean = 48.7, SD = 9.1), *p* = 0.015 (Fig. 2). The change in effect size for this item was in the medium range (Cohen’s *d* = 0.37).

Knowledge scores (out of a total of three questions) improved from 73 to 100%, although this was not statistically significant. There were trends toward significance in improvement with OUD pathophysiology and diagnosing OUD (*p* = 0.07). There was a modest non-significant improvement in managing OUD and no change observed with broad communication skills.

### Kirkpatrick Level 3

#### RQ2: Does the Intervention Increase Confidence in OUD Management Competencies?

Participants gained confidence in multiple domains, with statistically significant improvements in motivational interviewing and offering resources, with half of participants shifting from lacking confidence to confident (*p* = 0.04) (Table 1).

**Table 1:** Pre-intervention and Post-intervention Outcome Measures Divided by Survey Item. The MCRS Score Reflects Results from a Validated Construct Tool for Measuring Empathy. (*) Signifies Statistical Significance (*p* < 0.05). Confidence was Defined as Reporting “Moderately Confident,” “Very Confident,” or “Extremely Confident” *Knowledge Assessed via 3 Multiple-choice Questions (see Appendix 2)

Measure	Pre-intervention	Post-intervention	*p*-value	Effect size (*d*)
MCRS score	46.9 (9.0)	48.7 (9.1)	0.015*	0.37
*Knowledge score (≥ 2 of 3 correct)	11 (73.3%)	16 (100%)	-	-
Confidence in:	*n* (%) with confidence	*n* (%) with confidence		
Pathophysiology	7 (46.7%)	12 (75.0%)	0.07	-
Diagnosing OUD	7 (46.7%)	14 (87.5%)	0.07	-
Managing OUD	7 (46.7%)	9 (56.3%)	0.13	-
Communication	11 (73.3%)	12 (75.0%)	NA	-
Motivational interviewing	5 (33.3%)	12 (75.0%)	0.04*	-
Offering resources	4 (26.7%)	11 (68.8%)	0.04*	-

## DISCUSSION

This proof-of-concept pilot study demonstrates that patient-centered podcasts can improve provider attitudes toward patients with OUD and increase confidence in key clinical competencies. RQ1: We found a statistically significant improvement in attitudes toward patients with OUD as measured by MCRS (*p* = 0.015, Cohen’s *d* = 0.37). RQ2: Participants showed significant confidence gains in motivational interviewing and offering resources (*p* = 0.04), with trends toward improved confidence in OUD pathophysiology and diagnosis. RQ3: The innovation was highly acceptable and feasible, with 100% rating content as clear and credible, and 75% strongly agreed on ease of access.

Improvements in attitude after exposure to patient voice is consistent with data showing that patient involvement in curricula leads to a shift in focus to humanism and counters empathy decline^8^. While the 1.8-point MCRS improvement was statistically significant (*p* = 0.015), the clinical significance is uncertain because the MCRS lacks validated cutoffs on its 66-point scale. Larger studies are needed to determine what threshold translates to improved clinical behaviors and, ultimately, patient outcomes.

Several strategies have been described in the literature for teaching SUD to learners, such as role-playing with real-time coaching, which has shown efficacy in skill acquisition^11–15^. These strategies, however, all require significant time and logistical investment and do not address learner attitudes related to stigma. Studies show that the combination of education and direct contact with patients experiencing SUD is effective in reducing stigma ^16,17^.

### Implications

The results of our pilot suggest important implications for patient care. When learners engage with content that centers on the patient experience, this may foster improved provider-patient trust, encouraging patient engagement in change behaviors such as initiating SUD treatment or reducing substance use. If validated through larger studies, patient-centered podcasts could provide an easily implementable strategy within medical curricula to improve SUD patient outcomes by simultaneously addressing learner attitudes, knowledge, and behaviors through an accessible, asynchronous learning modality.

### Limitations

There are several limitations to highlight. Our needs assessment only includes individuals at a single academic institution and does not capture the perspectives of community practitioners. It also had a low response rate (10.9%) which, while containing trainee and gender diversity, may still limit the representativeness of identified educational gaps. Our needs, confidence, and knowledge assessments were internally developed and lack formal psychometric validation. Self-reported confidence may not reflect actual clinical competence or behavior. Our small sample size at a single site limits generalizability and the scope of our conclusions as well as meaningful subgroup analyses. But despite this, preliminary results demonstrating significant improvement in attitude scores warrant further investigation and should be interpreted as hypothesis-generating while demonstrating feasibility and preliminary efficacy.

Future studies should assess patient care impact (i.e. treatment engagement rates) and generalizability through multi-institutional randomized controlled trials comparing patient-centered podcasts to traditional education modules *(Kirkpatrick Level 4*). Standardized patient encounters with blinded assessment of communication skills, motivational interviewing could validate whether attitude changes translate to observable behavioral change. Longitudinal assessments at 6 and 12 months would determine durability of attitude and confidence improvements. We hope to develop faculty resources for incorporating these podcasts into residency curricula.

## CONCLUSION

In conclusion, using technology to incorporate patients’ lived experiences within medical education curricula could offer a cost-effective, scalable, and novel approach to promote humanism and improve attitudes toward marginalized populations among general internists.

## Supplementary Information

Below is the link to the electronic supplementary material.

ESM 1 (29.1 KB DOCX)

ESM 2 (27.9 KB DOCX)

ESM 3 (27.0 KB DOCX)

ESM 4 (300 KB DOCX)

ESM 5 (241 KB DOCX)

## Data Availability

The deidentified dataset supporting the findings of this study is available from the corresponding author upon reasonable request. Due to the small sample size (n=15) and potential identifiability of participants, data are not publicly available to protect participant privacy per IRB restrictions.
