# Supplementary material for: Patient-Centered Podcasts: An Educational Innovation to Improve Attitudes Toward Patients with Opioid Use Disorder Among Internal Medicine Practitioners
Source: J Gen Intern Med. 2026 Jan 29;41(7):1872–7. doi: 10.1007/s11606-026-10222-y (PMC13176433; doi:10.1007/s11606-026-10222-y)
Supplement: Supplementary file 1 — (29.1 KB DOCX) [file 11606_2026_10222_MOESM1_ESM.docx]

Appendix A: Needs Assessment Survey and Enrollment Invitation

We are excited to invite you to participate in an important project aimed at understanding and enhancing education around substance use disorders, including opioid use disorder (OUD). As a healthcare professional, your insights and

experiences are invaluable to us.

Phase 1: Needs Assessment

We are conducting a needs assessment to gather information about your current experience and education

regarding substance use disorders. Your participation will help us identify gaps and opportunities for improvement in

medical education. This survey will take you about < 7 minutes to complete.

Phase 2: Enrollment in Research Study

At the end of this survey, you will have the opportunity to enroll in a research study that involves listening to 3

patient-centered podcast episodes on OUD that are 30-40min in length on average. These podcasts are designed to

provide firsthand insights into the experiences of individuals with OUD, enhancing your understanding and empathy.

*Anonymity*:

In order to enroll you into the research project, we will request your contact information. However, all survey responses are anonymous, and no identifiable information is requested within the survey. Any free text options with responses will be reviewed and de-identified - meaning we will remove any personal information if added. Your

completion of the survey is your consent to participate..

*Incentive*:

Participation in the study will enter you into a raffle to win one of five $50 Amazon gift cards through random selection. Each podcast episode you listen to will increase your chances of winning.

Thank you for considering this opportunity to contribute to the advancement of medical education on substance use disorders. De-identified responses and data will be used in scholarly works such as publications and presentations. All results are anonymous and not linked to any individual. This study was reviewed and approved by the IRB of VUMC (240972).

This survey was developed to identify educational gaps in substance use disorder care among internal medicine providers. Items were designed to assess confidence across six core competency domains aligned with ACGME milestones for addiction medicine: (1) pathophysiology and neurobiology, (2) screening and diagnosis, (3) evidence-based treatment and medication-assisted treatment, (4) patient-centered communication, (5) motivational interviewing techniques, and (6) care coordination and resource provision.

1. Demographics
2. Gender: (M/F/Trans/Non-binary/Prefer not to answer)
3. Current level of training or employment :(M1/M2/M3/M4/R1/R2/R3/R4/Jr Faculty)
4. Please rate your level of confidence in each of the following areas pertaining to your own clinical care of patients with substance use disorders: (Not at all Confident/Slightly Confident/Somewhat Confident/Moderately Confident/Very Confident/Extremely Confident)
5. Screening
6. Obtaining substance use history
7. Motivational interviewing
8. Diagnosis
9. Medications for Opioid Use Disorder (MOUD) Treatment
10. Medications for Alcohol Use Disorder
11. Medications for Tobacco Use Disorder
12. Treatment of Stimulant Use Disorder
13. Referral to Services (Mutual Support, Rehabilitation, Substance Use Clinics, and Behavioral Health)
14. What aspects of treating patients with substance use disorders do you find most challenging? (Free Text Entry)
15. What type of learning format do you find most effective towards a better understanding of substance use disorders?
    1. Lecture
    2. Small Group
    3. Role Play
    4. Podcast
    5. Web-based Modules
    6. Journal Articles
    7. Other
16. Please describe your preferred learning format (Free Text Entry)
