# Supplementary material for: Patient-Centered Podcasts: An Educational Innovation to Improve Attitudes Toward Patients with Opioid Use Disorder Among Internal Medicine Practitioners
Source: J Gen Intern Med. 2026 Jan 29;41(7):1872–7. doi: 10.1007/s11606-026-10222-y (PMC13176433; doi:10.1007/s11606-026-10222-y)
Supplement: Supplementary file 2 — (27.9 KB DOCX) [file 11606_2026_10222_MOESM2_ESM.docx]

Appendix B: Validate Scales utilized in the Pre and/or Post surveys.

The **Toronto Empathy Questionnaire (TEQ)** consists of 16 statements designed to measure emotional empathy. Respondents indicate how frequently each statement applies to them using a 5-point scale: Never (0), Rarely (1), Sometimes (2), Often (3), and Always (4) for each item below.

1. When someone else is feeling excited, I tend to get excited too.
2. Other people's misfortunes do not disturb me a great deal.
3. It upsets me to see someone being treated disrespectfully.
4. I remain unaffected when someone close to me is happy.
5. I enjoy making other people feel better.
6. I have tender, concerned feelings for people less fortunate than me.
7. When a friend starts to talk about his/her problems, I try to steer the conversation to something else.
8. I can tell when others are sad even when they do not say anything.
9. I find that I am “in tune” with other people’s moods.
10. I do not feel sympathy for people who cause their own serious illnesses.
11. I become irritated when someone cries.
12. I am not really interested in how other people feel.
13. I get a strong urge to help when I see someone who is upset.
14. When I see someone being treated unfairly, I do not feel much pity for them.
15. I find it silly for people to cry out of happiness.
16. When I see someone being taken advantage of, I feel kind of protective towards them.

Negatively worded items (2, 4, 7, 10, 11, 12, 14, 15) are reverse scored. The total score reflects the respondent’s level of emotional empathy, with higher scores indicating greater empathy

The **Medical Condition Regard Scale (MCRS)** is a validated tool designed to assess healthcare providers’ attitudes, biases, and emotional responses toward patients with specific medical conditions. It is not condition-specific and can be adapted to various illnesses. The MCRS consists of 11 statements, each rated on a 6-point Likert scale from "strongly disagree" (1) to "strongly agree" (6). Some items are reverse-scored* to reduce response bias.

1. I feel especially compassionate toward patients like this.
2. Patients like this irritate me. (reverse scored)
3. I enjoy giving extra time to patients like this.
4. Treating patients like this is a waste of money.*
5. Patients like this are particularly difficult for me to work with.*
6. I can usually find something that helps patients like this feel better.
7. I prefer not to work with patients like this.*
8. Working with patients like this is satisfying.
9. I wouldn’t mind getting up on call nights to care for patients like this.
10. There is little I can do to help patients like this.*
11. Insurance plans should cover patients like this to the same degree that they cover patients with other conditions.

Higher total scores indicate a more positive regard for patients with the condition in question
