# Supplementary material for: Patient-Centered Podcasts: An Educational Innovation to Improve Attitudes Toward Patients with Opioid Use Disorder Among Internal Medicine Practitioners
Source: J Gen Intern Med. 2026 Jan 29;41(7):1872–7. doi: 10.1007/s11606-026-10222-y (PMC13176433; doi:10.1007/s11606-026-10222-y)
Supplement: Supplementary file 3 — (27.0 KB DOCX) [file 11606_2026_10222_MOESM3_ESM.docx]

Appendix C: Multiple-Choice Questions (MCQs) utilized for Knowledge Assessment

Three multiple-choice questions assessing knowledge of: (1) DSM-5 diagnostic criteria for OUD, (2) first-line medication-assisted treatment options, and (3) harm reduction strategies. Questions were developed based on current clinical practice guidelines and ASAM (American Society of Addiction Medicine) recommendations.

1) Which of the following is the final step in the neurobiology of addiction model?

1. Withdrawal and negative effect
2. Preoccupation and anticipation
3. Binge and intoxication
4. Cravings and Loss of control
5. Tolerance

2) Which medication used to treat opioid use disorder requires patients to travel to a designated clinic for administration?

1. Methadone
2. Buprenorphine
3. Naltrexone
4. All of the above
5. Methadone and Buprenorphine

3) In the context of treating opioid use disorder, the role of sponsors is primarily to:

1. Provide medication and medical treatment.
2. Offer practical advice, share personal experiences, and provide ongoing support.
3. Replace the role of healthcare providers in the patient's treatment.
4. Enforce strict abstinence and conduct regular drug tests on the patient.
5. Act as a care coordinator for patients' as they transition from rehab programs
