# Supplementary material for: Patient-Centered Podcasts: An Educational Innovation to Improve Attitudes Toward Patients with Opioid Use Disorder Among Internal Medicine Practitioners
Source: J Gen Intern Med. 2026 Jan 29;41(7):1872–7. doi: 10.1007/s11606-026-10222-y (PMC13176433; doi:10.1007/s11606-026-10222-y)

Appendix E: Surve-Items in Pre and Post Surveys Measuring Confidence in Diagnosis and Management of OUD.


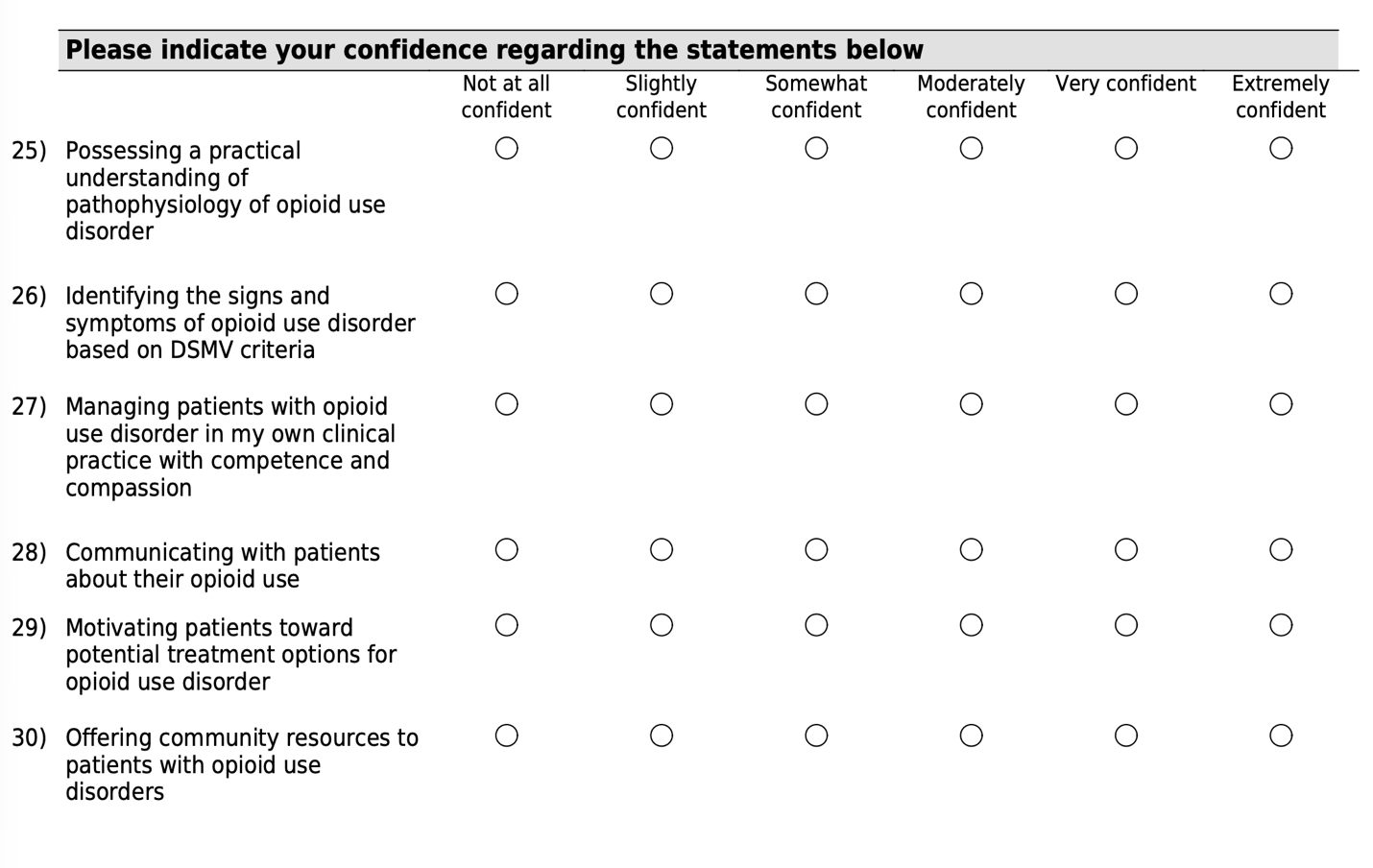

Supplement: Supplementary file 5 — (241 KB DOCX) [file 11606_2026_10222_MOESM5_ESM.docx]
